# The Effect of Medial Circumflex Artery Variation on the Success Rate of Gracilis Flap Procedures: A Systematic Review

**DOI:** 10.7759/cureus.73918

**Published:** 2024-11-18

**Authors:** Agathoklis Vallas, Dimosthenis Chrysikos, George Tsakotos, Theodore Troupis

**Affiliations:** 1 Anatomy, National and Kapodistrian University of Athens School of Medicine, Athens, GRC; 2 Anatomy and Surgical Anatomy: Orthopedic Surgery, National and Kapodistrian University of Athens School of Medicine, Athens, GRC; 3 Surgery, University of Athens Medical School, Athens, GRC

**Keywords:** anatomical variants, gracilis flap outcomes, medial circumflex artery variability, plastic and reconstructive surgery, reconstructive surgery techniques, vascular mapping

## Abstract

This systematic review examines the impact of anatomical variations in the medial circumflex artery (MCA) on the outcomes of gracilis flap procedures in reconstructive surgery. Incorporating 16 studies, this review analyzes how different MCA variants influence the success rates of these procedures. Findings highlight critical MCA variations, including cases of split and double pedicles and differences in pedicle length and branching patterns, which can complicate flap harvesting and vascular anastomosis. The studies consistently underscore the need for precise preoperative imaging and intraoperative adaptability to manage these anatomical differences effectively.

Notably, variants with shorter or branched pedicles present higher risks of partial flap loss and complications, particularly in autologous breast reconstruction and lower extremity repairs. The findings support the adoption of advanced imaging protocols, such as high-resolution Doppler and CT angiography, to enable detailed vascular mapping. Interdisciplinary collaboration among anatomists, radiologists, and surgeons is essential for developing comprehensive strategies tailored to individual anatomical landscapes, optimizing both the success and viability of gracilis flaps.

The review emphasizes that understanding MCA variability is crucial for enhancing surgical precision and improving patient outcomes. Standardizing preoperative assessment protocols and exploring alternative flap techniques may mitigate risks associated with MCA variations, advancing the field of reconstructive surgery.

## Introduction and background

In the realm of reconstructive surgery, the quest for optimal outcomes following tissue loss due to oncological resections, trauma, or congenital defects remains a pivotal challenge. Among the myriad options available to reconstructive surgeons, the gracilis muscle flap stands out due to its versatility, reliability, and relatively straightforward harvesting procedure. Originating from this arena is a nuanced topic that addresses the relationship between the anatomical variations of the medial circumflex artery (MCA) and the success rates of gracilis flap procedures. This topic is not only a reflection of the intricate interplay between anatomy and surgical outcomes but also underscores the evolution of microsurgical techniques and their impact on patient care [1]. 

The gracilis muscle, nestled within the medial compartment of the thigh, has long been favored for its utility in both functional reconstruction and aesthetic repair. Its role is critically underscored in the reconstruction of soft tissue defects, particularly in breast reconstruction post-mastectomy, facial reanimation following paralysis, and coverage of perineal and lower extremity defects. The inherent advantages of the gracilis flap, including its consistent vascular supply, minimal donor site morbidity, and adaptable muscle bulk, have established it as a mainstay in the armamentarium of reconstructive options [2]. 

Central to the success of gracilis flap procedures is the MCA, which predominantly supplies blood to the gracilis muscle. The MCA, with its branches, plays a critical role in ensuring the viability and successful integration of the gracilis flap at the recipient site. However, the anatomy of the MCA is marked by significant variability, with differences noted in its origin, size, branching pattern, and relationship to neighboring structures. These variations can present considerable challenges during flap harvest, impacting not only the technical aspects of the procedure but also the ultimate success of the reconstruction [3]. 

The intricacies of the MCA’s anatomy and its implications for gracilis flap surgeries have been the subject of several studies, with researchers seeking to delineate patterns, predict variations, and devise strategies to mitigate their impact on surgical outcomes. Despite these efforts, the variable nature of the MCA continues to command respect and caution from surgeons, highlighting the importance of a deep anatomical understanding and meticulous surgical planning [3]. 

Aim and research questions 

The overarching objective of this comprehensive review is to meticulously examine and synthesize existing research regarding the impact of medial circumflex artery (MCA) variations on the success rates of gracilis flap procedures. This examination is paramount, as the MCA's variability can significantly influence the outcomes of these complex reconstructive surgeries. By collating and critically appraising the findings from a wide array of studies, this review aims to shed light on the intricate relationship between anatomical diversity and surgical success, thereby providing a consolidated resource for surgeons, researchers, and educators alike. 

Furthermore, this review seeks to identify and dissect the commonalities and discrepancies within the existing literature, offering a nuanced understanding of the factors that contribute to the efficacy of gracilis flap procedures. By analyzing studies from different geographical regions and patient demographics, this paper endeavors to offer a global perspective on the subject, recognizing the influence of anatomical diversity across populations. Another essential purpose of this review is to uncover gaps in the current body of knowledge, highlighting areas in need of further research and investigation. This not only includes the anatomical and surgical realms but also extends to patient outcomes, satisfaction, and the long-term viability of reconstructive efforts. Identifying these gaps is a critical step toward advancing the field and guiding future research endeavors. 

Ultimately, this review aims to equip surgeons and medical professionals with a deeper understanding of the challenges and considerations associated with gracilis flap procedures influenced by MCA variations. By providing a comprehensive overview of existing findings coupled with a critical analysis of their implications, this document intends to support informed decision-making in clinical practice. It aspires to contribute to the enhancement of surgical techniques, preoperative planning, and patient care, thereby improving the overall success rates of reconstructive surgeries utilizing the gracilis flap. This endeavor reflects a broader commitment to advancing the field of reconstructive surgery through meticulous scholarship and evidence-based practice. 

The aim of this study is to systematically review the impact of MCA anatomical variations on the outcomes of gracilis flap procedures in reconstructive surgery. Specifically, this review seeks to identify and characterize key MCA variants that may influence the success rates of these procedures, highlighting challenges and considerations in surgical planning and execution. The following research questions guide this investigation: What are the main anatomical variations of the MCA relevant to gracilis flap procedures, and how do they impact surgical outcomes; Which MCA variants are most frequently associated with complications or compromised success rates in gracilis flap procedures; What surgical techniques and preoperative planning strategies are recommended to accommodate MCA variability and optimize patient outcomes? 

The significance of this topic extends beyond the technicalities of surgical procedures. It delves into the heart of patient-centered care, addressing the balance between achieving optimal reconstructive outcomes and minimizing the risk of complications. In the context of breast reconstruction, for instance, the successful integration of a gracilis flap can profoundly affect a patient's psychological recovery, body image, and overall quality of life post-mastectomy. Similarly, in the realm of facial reanimation or lower extremity reconstruction, the functional and aesthetic outcomes can significantly influence patient satisfaction and long-term well-being [4]. 

Moreover, the discussion surrounding the MCA and gracilis flap procedures is reflective of broader themes within reconstructive surgery and medicine at large. It embodies the ongoing pursuit of precision medicine, tailored to the individual's specific anatomical and physiological landscape. It also underscores the importance of interdisciplinary collaboration, combining insights from anatomy, surgery, radiology, and patient care to enhance surgical outcomes. Furthermore, this topic serves as a window into the evolving nature of surgical education and training. As the understanding of MCA variability and its implications for gracilis flap procedures deepens, so too does the need for comprehensive educational models that prepare future surgeons for the complexities they may encounter. This encompasses not only the mastery of anatomical knowledge and surgical techniques but also the development of critical thinking and problem-solving skills essential for navigating unpredictable scenarios [4]. 

The Importance of the Medial Circumflex Artery in Gracilis Flap Procedures 

The medial circumflex artery (MCA) holds a pivotal role in the realm of reconstructive surgeries, particularly within the context of gracilis flap procedures. This artery's significance is deeply rooted in its responsibility for supplying blood to the gracilis muscle, an aspect that dictates the success and viability of flap transplants. The centrality of the MCA in such operations can be traced back to the foundational principles of reconstructive surgery, where the primary objective is to restore form and function with minimal complications [5]. 

In the specialized procedure utilizing the gracilis muscle, the MCA not only supplies the necessary vascular support but also ensures the endurance and integration of the transplanted tissue into its new environment. The unique anatomical positioning and vascular pattern of the MCA make it an artery of choice for surgeons looking to harness the gracilis muscle's potential in reconstructive efforts. Its significance is further accentuated in cases requiring delicate microvascular anastomoses, where the artery's caliber and flow characteristics become critical determinants of success [5]. 

The gracilis flap, primarily served by the MCA, is renowned for its flexibility and reliability in covering defects, particularly in breast reconstruction, facial reanimation, and lower extremity reconstructions. The artery's ability to provide a consistent blood supply underpins the flap's healing and integration post-transplantation. However, the MCA's anatomical variations present a unique set of challenges that demand a thorough understanding and meticulous planning. These variations can significantly impact the surgical approach, necessitating modifications to the standard procedural techniques to accommodate the individual patient's vascular anatomy [6]. 

Moreover, the MCA's relevance extends beyond the immediate surgical outcomes. The artery's health and integrity are crucial for the long-term viability of the gracilis flap. Compromises in its blood supply can lead to complications such as flap failure, tissue necrosis, or prolonged healing times, thereby affecting the overall success of the reconstructive effort. Consequently, preoperative assessments, including vascular imaging and mapping, become indispensable tools in understanding the MCA's pathway and preparing for potential anomalies [7]. 

The importance of the MCA in gracilis flap procedures encapsulates a broader narrative within reconstructive surgery that emphasizes the integration of detailed anatomical knowledge with surgical skills. Understanding the MCA's nuances not only facilitates the technical execution of gracilis flap transplants but also influences the strategic decisions that guide patient-specific reconstructive plans. This intricate interplay between anatomy and surgical technique underscores the MCA's central role in the successful application of gracilis flap procedures, highlighting its importance as not merely a vessel of blood but as a lifeline that determines the restorative potential of the gracilis muscle in the diverse landscape of reconstructive surgery [6]. 

Anatomy and Physiology of the Medial Circumflex Artery 

The embryological development of the medial circumflex artery (MCA) is integral to understanding its functional significance and variability in gracilis flap surgeries. The MCA originates embryologically from the lateral femoral circumflex system, which itself develops from the primitive axial artery. This embryonic artery represents a precursor to the major arteries of the lower limb, including the femoral artery, from which the profunda femoris artery and subsequently the MCA derive [8]. 

During the development of the lower limb in the fetus, the MCA emerges in conjunction with the differentiation and growth of the musculature of the thigh. The artery's course and branching pattern are influenced by the migration and maturation of the surrounding muscle groups, notably the adductor and quadriceps muscles, which it will eventually supply. This relationship underscores the crucial role of the MCA in not only providing vascular support to the thigh muscles but also in determining the successful integration and functionality of the gracilis muscle post-transplant [9]. 

The precise anatomical trajectory and branching of the MCA are established during the later stages of fetal development, and variability in these aspects can be traced back to differences in embryonic vascular formation. These variations are significant because they affect the surgical planning and outcome of flap procedures. Understanding the embryological origin and development of the MCA aids surgeons in anticipating and managing anatomical variations that could impact the viability of the gracilis flap [10]. 

The medial circumflex artery (MCA) plays a pivotal role in the landscape of flap surgery, particularly within the realm of reconstructive procedures utilizing the gracilis muscle. This essential vascular conduit not only orchestrates the blood supply to the upper medial thigh region but also underpins the viability and successful integration of the gracilis flap in various reconstructive endeavors. Delving into the anatomy and physiology of the MCA unveils the complexities and intricacies that underscore its critical function in flap surgery [11]. 

Originating from the profunda femoris artery, which itself is a major branch of the femoral artery, the MCA embarks on its course by traveling posteriorly, navigating through the intricacies of the thigh's muscular and fascial layers. The artery typically emanates from the lateral side of the profunda femoris artery, but variations in its origin are not uncommon, reflecting the individual nuances that can influence surgical strategies and outcomes. As it progresses, the MCA intricately weaves between the adductor and quadriceps muscles, a path that underscores its significance and the meticulous dissection required during surgical harvest [11]. 

The MCA’s branching pattern is equally complex, giving rise to several collateral branches that supply blood to the surrounding muscles, including the adductor, hamstring, and quadriceps groups. This extensive vascular network not only ensures the metabolic needs of the thigh's musculature but also establishes the MCA as a critical player in the perfusion of the gracilis muscle. The artery's terminal branches, particularly those permeating the gracilis muscle, are of paramount importance in flap surgeries, as they dictate the vascular viability of the harvested tissue [12]. 

In the context of flap surgery, the MCA’s significance is multifaceted. Firstly, the artery's vascular territory encompasses the gracilis muscle, making it the lifeline for gracilis flap procedures. The success of such surgeries hinges on the meticulous preservation of the MCA’s branches that specifically supply the gracilis muscle. These branches, characterized by their size, path, and branching patterns, are crucial for ensuring adequate perfusion and subsequent survival of the transplanted flap. Secondly, the anatomical trajectory and branching pattern of the MCA bears direct implications on surgical planning and execution. Surgeons must navigate the variability in the MCA’s anatomy with precision, as deviations from the norm can impact the dissection process, the length of the vascular pedicle, and ultimately, the success of the flap transfer. This necessitates a comprehensive preoperative assessment, often involving advanced imaging techniques, to map the MCA’s course and its branches. Such meticulous planning is imperative to mitigate the risks of inadvertent vascular injury, inadequate perfusion, and flap failure [13]. 

Furthermore, the physiology of the MCA, particularly its role in regulating blood flow to the gracilis muscle, highlights its importance in the postoperative period. The artery’s ability to adapt to the increased demands of the transplanted tissue and maintain adequate perfusion is crucial for flap integration and healing. This adaptive capacity, however, can be influenced by various factors, including the patient's vascular health, the integrity of the anastomoses, and the presence of systemic conditions that may impair blood flow [13]. 

The relationship between the MCA and the gracilis muscle extends beyond mere anatomy and physiology; it encompasses the principles of flap viability and the dynamics of tissue perfusion. In gracilis flap surgeries, the preservation of a robust vascular pedicle, primarily composed of the MCA and its venae comitantes, is instrumental. This pedicle not only facilitates the microvascular anastomosis to the recipient site but also serves as the conduit for the necessary nutrients and oxygen pivotal for the survival of the transplanted tissue. Moreover, the challenges encountered in preserving the MCA during gracilis flap harvest reflect the delicate balance between surgical intervention and the preservation of tissue viability. Surgeons must tread carefully, balancing the need for adequate pedicle length and diameter against the imperative of minimizing trauma to the vascular structure. This balance is critical in ensuring the success of the flap procedure, emphasizing the need for a deep understanding of the MCA's anatomy and physiology [14]. 

Gracilis Flap Procedures 

Gracilis flap procedures represent a significant advancement in the field of reconstructive surgery, providing solutions for a variety of complex clinical scenarios. The procedure, which involves the harvest and transfer of the gracilis muscle from the thigh, has become an essential technique for surgeons aiming to restore form and function to various parts of the body affected by trauma, congenital defects, or disease processes such as cancer [2]. 

The gracilis muscle, located in the medial compartment of the thigh, is chosen for its favorable anatomy and physiology. It is a long, thin muscle, which makes it easily adaptable to different reconstructive needs. The muscle’s vascular supply primarily comes from the medial circumflex femoral artery, which allows for a reliable blood supply to the muscle and overlying skin, making it an excellent candidate for free flap procedures. This feature is particularly crucial as it determines the viability and successful integration of the flap into the recipient site [15]. 

Gracilis flap procedures are utilized in a myriad of reconstructive scenarios. In breast reconstruction, the gracilis flap, often harvested with a skin paddle, provides an autologous tissue option for women undergoing mastectomy due to breast cancer. This approach offers a less invasive alternative to the more extensive abdominal flap procedures, with the added benefit of reducing donor site morbidity and preserving abdominal muscle strength. Similarly, in the realm of facial reanimation, segments of the gracilis muscle are used to restore movement to facial features paralyzed by conditions such as Bell's palsy or post-surgical complications. This application underlines the muscle's versatility and capacity to integrate and function in different anatomical regions [16]. 

Furthermore, the gracilis flap has been instrumental in reconstructing soft tissue defects in the extremities and the perineal region. For patients suffering from soft tissue loss due to trauma, infection, or oncologic resections, the gracilis flap can provide essential coverage, helping to restore not only the physical integrity but also preventing further complications such as infection. In cases of lymphedema, segments of the gracilis muscle have been used to create pathways for lymphatic drainage, showcasing the innovative applications of this flap beyond mere defect coverage [2]. 

The significance of gracilis flap procedures in reconstructive surgery cannot be overstated. They offer a balance between morbidity and functionality, providing tissue that can adapt to various contours and functional needs. The procedure underscores the principles of reconstructive surgery: to replace "like with like," offering tissues similar in texture and function to the areas they are meant to reconstruct. The relative ease of harvest and the muscle's consistent vascular anatomy further contribute to the flap's popularity among reconstructive surgeons [17]. 

However, the success of gracilis flap procedures hinges on meticulous surgical technique and a deep understanding of the muscle’s vascular supply, particularly the variations in the medial circumflex femoral artery. Preoperative planning involves detailed imaging to map out the vascular anatomy and to plan the flap harvest accordingly. During the procedure, surgeons must carefully dissect the muscle to preserve the vascular pedicle, ensuring the viability of the transferred tissue. Postoperative care, too, plays a critical role, with close monitoring for signs of compromised blood supply to the flap, which could necessitate urgent intervention [18]. 

The evolution of gracilis flap procedures reflects broader trends in reconstructive surgery toward less invasive techniques, patient-specific customization, and multidisciplinary care. As surgical techniques and preoperative planning have advanced, so too have the outcomes and possibilities for patients requiring reconstruction. The procedure embodies the fusion of art and science that defines plastic and reconstructive surgery, an intricate understanding of anatomy combined with a creative approach to solving complex reconstructive challenges [18]. 

The gracilis flap procedure is a testament to the ingenuity and innovation inherent in reconstructive surgery. Its applications across different regions of the body highlight its versatility and adaptability. The procedure not only addresses the aesthetic and functional deficits but also significantly contributes to the overall well-being and quality of life of patients. As the understanding of the gracilis muscle’s vascular supply and the technical aspects of the procedure continue to evolve, so too will the scope and success of gracilis flap reconstructions in the ever-advancing field of reconstructive surgery. 

Materials and methods

**Figure 1:**
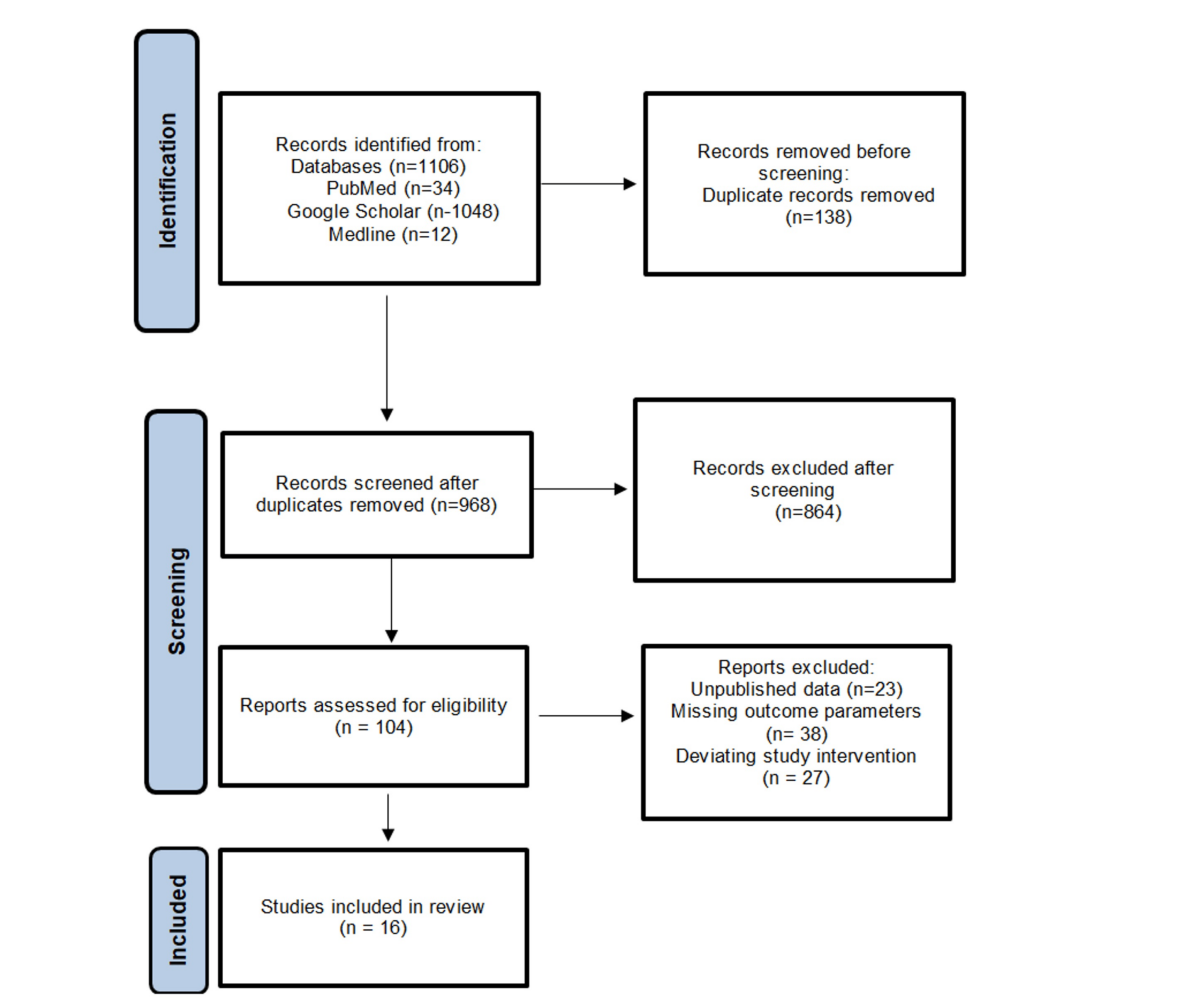
PRISMA analysis flow chart. PRISMA: Preferred Reporting Items for Systematic Reviews and Meta-Analyses

This systematic review was performed in accordance with the Preferred Reporting Items for Systematic Reviews and Meta-Analyses (PRISMA) guidelines. The protocol of this systematic review has been submitted to the Institutional Review Board of the Department of Anatomy, National and Kapodistrian, University of Athens, Greece, and is available upon request. Eligible articles were identified by a search of Medline, PubMed, and Google Scholar bibliographical databases for the period from 24/05/2024 up to 24/06/2024. The study protocol was agreed upon by all co-authors. The search strategy included the following keywords: ("Medial Circumflex Artery" OR "MCA") AND ("gracilis flap" OR "gracilis muscle flap") AND ("surgical outcomes" OR "surgical success" OR "reconstructive outcomes") AND ("anatomical variations" OR "anatomical diversity") AND ("reconstructive surgery") AND ("patient outcomes" OR "patient satisfaction" OR "long-term viability"). Language restrictions were applied (only articles in English, French, and German were considered eligible); two investigators (AV and DC), working independently, searched the literature and extracted data from each eligible study. Reviews were not eligible, while all prospective and retrospective studies, as well as case reports, were eligible for this systematic review. Manuscripts that did not state the names of the authors were excluded. In addition, we checked all the references of relevant reviews and eligible articles that our search retrieved, so as to identify potentially eligible conference abstracts. Titles of interest were further reviewed by abstract. Finally, reference lists of eligible studies were manually assessed in order to detect any potentially relevant article (“snowball” procedure). The PRISMA flowchart of this systematic review is presented in Figure 1, illustrating the identification, screening, and inclusion processes of the studies reviewed. 

## Review

Results

Summary of Articles 

A total of 16 studies were included in this systematic review to assess the impact of medial circumflex artery (MCA) variability on the success rates of gracilis flap procedures. Table 1 provides a comprehensive summary of these studies, outlining the purpose, methodology, and key results of each. The included studies encompass various aspects of gracilis flap procedures, ranging from technical innovations to anatomical variations and their clinical implications. This summary highlights the diverse approaches and findings in the existing literature, emphasizing the critical role of meticulous preoperative planning and intraoperative adaptability to manage the challenges posed by MCA variability effectively. 

**Table 1:** A brief presentation of the studies included in the systematic review. TUG: transverse upper gracilis; MCFA: medial circumflex femoral artery; FFMT: functioning free muscle transfer; MCFAP: medial circumflex femoral artery perforator; BCS: breast-conserving surgery

Authors/Year	Purpose of Study	Study Methodology	Key Study Results
Azizzadeh and Pettijohn, 2016 [2]	To evaluate the efficacy of the gracilis free flap in dynamic facial reanimation using a 2-stage procedure.	Retrospective review of patients undergoing gracilis free flap for facial reanimation; single surgeon; 2-stage procedure.	High success in facial reanimation, and low flap failure rate. Significant improvement in facial symmetry and smile excursion. Some patients required a third stage for refinement.
Magden et al., 2010 [5]	To provide precise information about the major and minor vascular pedicles and innervation of the gracilis muscle.	Dissection of the gracilis muscles in 15 formalin-fixed adult cadavers (30 cases) with 4x loupe magnification, and latex injection through the external iliac artery.	The most proximal pedicle (deep branch of the medial circumflex femoral artery) was located 60 mm from the pubic tubercle with a mean diameter of 0.9 mm. The second pedicle (medial circumflex femoral artery) was dominant in 13% of cases, with a mean diameter of 1.2 mm. The major pedicle was the deep femoral artery, dominant in 87% of cases, with a mean diameter of 1.6 mm. Distal pedicles from the superficial femoral artery were present in all cases, double in 77%, with a mean diameter of 1.4 mm. The anterior branch of the obturator nerve was the motor nerve in all cases. The findings suggest that the middle part of the gracilis muscle could be elevated as a free flap based on the distal pedicles.
Natoli and Wu, 2015 [19]	To report experience with pedicle variability in the transverse upper gracilis (TUG) myocutaneous flap for autologous breast reconstruction.	Retrospective review of 36 TUG flaps performed on 24 patients by a single surgeon between July 2006 and November 2011.	17% of cases exhibited pedicle variability: 5.5% had a split pedicle and 11% had a double main pedicle. One partial flap loss and four cases of lymphedema.
Shibuya et al., 2017 [20]	To evaluate the anatomic basis of the medial circumflex femoral artery (MCFA) perforators from the medial circumflex femoral vessels and assess clinical outcomes.	Examined 55 breast reconstruction procedures using MCFA perforator flaps between July 2010 and June 2014. Recorded number, location, and distance of perforators intraoperatively.	Identified 131 MCFA perforators (mean 2.4 per patient). Majority of perforators coursed through the gracilis muscle. Mean flap weight was 202g. No major complications. Partial fat necrosis in two cases.
Lasso et al., 2004 [21]	To report an anatomical variation of the main vascular pedicle of the gracilis muscle.	Case report of a middle-aged woman undergoing gracilis muscle flap surgery.	Identified an anatomical variation where the pedicle penetrates the fascia of the adductor longus and runs between its muscle fibers, complicating dissection.
Arnež et al., 2004 [22]	To evaluate the effectiveness of the transverse upper gracilis (TUG) flap for breast reconstruction.	Case series of seven patients who underwent breast reconstruction using the TUG flap in 2002.	Five out of seven flaps survived; two flaps were lost in the same patient. Complications included wound dehiscence on the thigh. TUG flap is suitable for women with small or moderately large breasts seeking primary autologous reconstruction.
Hattori et al., 2002 [23]	To describe a vascular dissection technique for an easy and safe approach to the vascular pedicle of the gracilis muscle for functioning free muscle transfer (FFMT).	Detailed description of a surgical technique for dissecting the gracilis muscle's vascular pedicle. Retrospective review of 100 cases and cadaveric dissection.	The technique allows for safe and easy dissection of the gracilis pedicle, providing adequate length and diameter for microvascular anastomosis.
Izumi et al., 2013 [24]	To evaluate the effectiveness of free medial circumflex femoral artery perforator (MCFAP) flaps for immediate breast reconstruction after breast-conserving surgery (BCS).	Case series of 15 patients who underwent immediate breast reconstruction using free MCFAP flaps between April 2012 and January 2013.	Mean flap weight was 138.5 g, mean pedicle length was 5.7 cm. No major complications requiring surgical intervention. Partial fat necrosis in two patients, and wound infection in one patient. High satisfaction with cosmetic outcomes.
Kappler et al., 2005 [25]	To analyze the proximal perforator vessels of the gracilis musculocutaneous flap.	Dissected 23 cadaver legs preserved by Thiel method; injected with red silicone mass. Ink-injected and clarified additional cadaver legs.	Found considerable variation in number and localization of perforators. One to four perforators with diameters ranging from 0.5 to 1.0 mm were found within a 6x6 cm area. The average perfused skin area was 88 cm². Variability in perforator anatomy suggests the flap can be used by experienced surgeons, but the anatomy is unpredictable.
Hallock, 2003 [26]	To evaluate the effectiveness of the medial circumflex femoral GRACILIS perforator flap for groin wounds.	Retrospective review of four patients with infected groin wounds; combined conjoint flap with gracilis muscle.	Uncomplicated healing in all cases; no recurrent infections. One case of venous congestion resolved with leech therapy. Direct donor site closure was possible in all cases.
Hallock, 2006 [27]	To evaluate the medial circumflex femoral artery perforator (MCFAP) flap for scrotal reconstruction after Fournier gangrene.	Case report of a 68-year-old male with Fournier gangrene; use of MCFAP flap for neoscrotum creation.	Successful reconstruction with no major complications; minor issues such as neoscrotal hematoma, partial flap necrosis, and tethering to the thigh were managed conservatively.
Lykoudis et al., 2005 [28]	To perform a detailed study of the anatomy of the gracilis perforator flap.	47 dissections were performed in cadavers and clinical cases. Anatomical dissection and Doppler evaluation followed by surgical dissection.	At least one large calibre musculocutaneous perforator was found in 87% of dissections. Most perforators were located within a 7 cm radius from the entry point of the main pedicle. Methylene blue injections confirmed skin perfusion over the proximal third of the muscle. The presence of a superficial vein and occasional sensory nerve was noted.
Hallock, 2004 [29]	To demonstrate the use of the conjoint medial circumflex femoral perforator-gracilis muscle flap.	Case series of four conjoint flaps used to treat three patients with traumatic extremity wounds.	All flaps survived with minimal complications. The study highlights the flap's reliability, consistent vascular pedicle, and easy concealment of donor-site scars.
Hasen et al., 2003 [30]	To describe a novel technique to improve the dissection of the proximal gracilis vascular pedicle.	Retrospective review of 18 patients who underwent gracilis muscle free flap harvesting using the modified technique. Cadaveric dissection was also performed.	100% flap survival rate. One minor complication (seroma) and one major complication (venous thrombosis, flap salvaged). Technique allows safe and easy dissection with excellent observation of the proximal pedicle.
Eom et al., 2011 [31]	To evaluate the effectiveness of the upper medial thigh perforator flap for lower extremity reconstruction.	Case series of 40 patients with lower extremity soft-tissue defects who underwent reconstruction using this flap.	39 out of 40 flaps survived. Average flap size was 71.6 cm², with 90% originating from the medial circumflex femoral artery. Reliable for reconstructing lower extremities despite short pedicle length and small vessel diameter.
Hallock, 2004 [32]	To evaluate the medial circumflex femoral gracilis (MCFGRACILIS) perforator free flap.	Case series of seven MCFGRACILIS perforator free flaps in six patients, mostly for trauma-related defects.	Six out of seven flaps survived with minimal complications. The flap offers reliable skin territory, avoids muscle sacrifice, and has a consistent vascular pedicle.

Variations of the Medial Circumflex Artery 

The anatomical variations of the medial circumflex artery (MCA) and their implications for gracilis flap procedures have been the focus of several recent studies, each contributing valuable insights into the complexity and variability inherent in this area of the human anatomy. Notable among these are the findings from Natoli and Wu [19], Shibuya et al. [20], and Lasso et al. [21], which together offer a comprehensive overview of the challenges and considerations in utilizing gracilis flaps for reconstructive purposes, particularly in breast reconstruction. 

The study conducted by Natoli and Wu [19] reveals significant variability in the vascular pedicle associated with the transverse upper gracilis (TUG) myocutaneous flap, a modification aimed at improving outcomes in autologous breast reconstruction. Their retrospective review highlighted considerable variation in pedicle anatomy, with anomalies observed in 17% of cases. Specifically, they reported a 5.5% incidence (two cases) of a split pedicle and an 11% incidence (four cases) of a double main pedicle. Notably, one case of partial flap necrosis was documented, though it was not associated with these particular anatomical anomalies. These findings underscore the underestimated complexity of pedicle variability and its potential implications for flap viability and patient outcomes, including risks such as partial flap loss and lymphedema. This study also contributes a logical algorithm for flap dissection to address such variability, emphasizing the importance of careful evaluation and handling of vascular pedicles to ensure successful microsurgical breast reconstruction. 

Shibuya et al. [20], on the other hand, explore the anatomic basis of the medial circumflex femoral artery perforators (MCFAp) in the context of fascioadipocutaneous flap procedures for breast reconstruction, particularly relevant for Asian women with smaller breast sizes. Their findings, based on 55 flap procedures, indicate a mean of 2.4 MCFA perforators, predominantly passing through the gracilis muscle. The study also highlights the importance of perforator mapping for effective intraoperative identification of MCFA perforators, which can significantly impact the choice between MCFAp and posterior medial thigh perforator flaps. This research underlines the need for a thorough anatomical understanding and careful planning in utilizing medial thigh flaps for breast reconstruction, ensuring optimal outcomes with minimal donor site morbidity. 

Lasso et al. [21] present a unique perspective by reporting on an anatomical variation in the vascular pedicle of the gracilis muscle observed during a surgical procedure. Their report emphasizes the anatomic consistency typically associated with the gracilis muscle's vascular pedicle, yet they highlight a particular case where unexpected variation presented a surgical challenge. The variation they observed involved the pedicle penetrating the fascia of the adductor longus muscle, an anomaly that required adapted dissection techniques and prolonged the surgical time. This case illustrates the potential for anatomical variations to complicate otherwise routine procedures and stresses the need for vigilance and adaptability in surgical planning and execution. 

Collectively, these studies underscore the complex and variable nature of the vascular anatomy associated with the gracilis muscle and its implications for reconstructive surgery. The findings from Natoli and Wu [19] and Shibuya et al. [20] contribute to a growing body of evidence suggesting that while gracilis flaps offer valuable options for autologous breast reconstruction, their success is heavily dependent on the surgeon’s understanding of and ability to adapt to vascular anomalies. Moreover, the case reported by Lasso et al. [21] serves as a reminder of the unpredictable nature of human anatomy and the need for preparedness for anatomical variations. These insights collectively highlight the importance of detailed preoperative planning, intraoperative flexibility, and the development of surgical strategies to accommodate the wide range of anatomical variability in the medial circumflex artery and its branches. This understanding is crucial for improving the success rates of gracilis flap procedures and ensuring optimal patient outcomes. 

The study by Arnež et al. [22] offers a comprehensive exploration of the transverse upper gracilis (TUG) flap, illuminating both its potential and limitations as a resource in reconstructive breast surgery. This particular examination delves into the anatomical, procedural, and postoperative dimensions of employing the TUG flap for breast reconstruction, a subject that holds significant relevance given the evolving landscape of autologous reconstruction preferences among patients and surgeons alike. 

The TUG flap, as detailed by Arnež et al. [22], represents a segment of the gracilis muscle paired with a transversely oriented skin paddle, primarily nourished by the ascending branch of the medial circumflex femoral artery. Notably, the study underscores the flap's suitable dimensions and the relatively short but adequate vascular pedicle, which, despite its length, facilitates anastomosis with the internal mammary vessels. This anatomical synergy is particularly highlighted, underscoring the natural compatibility between the flap’s vascular pedicle and the recipient site’s vessels, an aspect that presumably contributes to the simplification of the microvascular process. 

In their 2004 cohort, Arnež et al. [22] reveal a mixed outcome: while the majority of the procedures were successful, instances of flap loss were observed. However, these cases of flap loss were not directly associated with MCA variations but rather with other factors, such as issues in vascular anastomosis and postoperative complications. This finding underscores the inherent risks in free flap breast reconstructions and invites a discussion on the meticulous surgical planning and execution required in TUG flap procedures. The complications mentioned notably thrombosis and wound dehiscence, especially when the flap width exceeds 10 cm, highlight the critical balance between flap size and patient anatomy. The insight into wound dehiscence emphasizes the surgical challenges and postoperative care that must be addressed when the TUG flap is employed, particularly focusing on the impact of flap dimensions on healing outcomes. 

The selection criteria delineated by Arnež et al. [22] for TUG flap candidacy-focusing on patients who are unsuitable or unwilling to undergo abdominal-based reconstructions highlight a significant patient-centered approach in reconstructive surgery. This approach aligns with current trends in patient autonomy and tailored surgical strategies, emphasizing a preference for minimizing donor site morbidity while optimizing aesthetic outcomes. However, the specificity of these criteria also points to the limitations of the TUG flap, notably in the reconstruction of large or ptotic breasts, thus underscoring the necessity for careful patient selection and surgical planning. 

The discussion on the strategic choice of recipient vessels, favoring the internal mammary vessels over axillary ones due to the pedicle length, provides critical surgical insights. The complications arising from forced deviation from this standard, as in the case of thrombosed internal mammary vessels requiring vein grafts, highlight the complexities and potential for increased complication rates when the ideal anatomical setup is altered. Arnež et al.’s [22] emphasis on the TUG flap's plasticity and its consequent aesthetic benefits in suitable cases enriches the narrative on its applicative advantages. The comparative discussion positioning the TUG flap against abdominal and gluteal alternatives sheds light on the nuanced decision-making process in flap selection, weighing factors such as volume, donor site morbidity, and aesthetic outcomes. However, while the study presents a valuable case series and reflections on the TUG flap’s utility, it inadvertently highlights a gap in the literature and a broader discussion on the long-term outcomes, patient satisfaction, and functional impacts of harvesting the gracilis muscle. Additionally, the absence of a comparative analysis with other flap types in terms of patient-reported outcomes leaves a void in understanding the full spectrum of patient experiences following TUG flap reconstruction. 

Hattori et al. [23] focus on the technical aspects of harvesting the gracilis muscle for functioning free muscle transfer (FFMT), particularly for brachial plexus reconstruction. They highlight the necessity of obtaining a longer vascular pedicle in FFMT compared to pedicled island transfer and detail an innovative endoscopic technique for safe and efficient vascular dissection. Their technique, which involves both anterior and posterior retraction of the adductor longus muscle, is designed to maximize the length and diameter of the vascular pedicle, thereby enhancing the success rates of FFMT by ensuring adequate vascular supply for microvascular procedures. The authors emphasize the importance of tracing the vascular pedicle to its origin from the profunda femoris vessels to secure a pedicle of sufficient length and diameter, reflecting the critical role of detailed anatomical understanding in improving FFMT outcomes. 

Izumi et al. [24] report on the use of medial circumflex femoral artery perforator (MCFAP) flaps for immediate breast reconstruction after breast-conserving surgery, underscoring the flap's reliability and minimal donor-site morbidity. Their findings demonstrate the efficacy of MCFAP flaps in achieving satisfactory cosmetic outcomes with minimal complications, including isolated cases of partial flap necrosis and local wound issues, both of which were resolved without surgical intervention. Notably, these instances of partial necrosis were not specifically linked to MCA variations but rather to general vascular challenges in flap perfusion. The study reports no instances of donor-site seroma or lymphedema, highlighting the advantages of MCFAP flaps in preserving donor-site functionality and aesthetics. However, the authors also acknowledge potential limitations, such as the relatively smaller available flap volume and the shorter pedicle length compared to other flaps. Despite these challenges, the positive outcomes reported in this study support the utility of MCFAP flaps in partial breast reconstruction, particularly for patients with small to medium-sized breasts. 

Anatomical Variations and Clinical Implications of Gracilis Flap Procedures 

The anatomical variations of the gracilis muscle and its vascular pedicles have significant implications for reconstructive surgery, as demonstrated by various studies. Collectively, these studies underscore the complexity and variability in the vascular anatomy associated with the gracilis muscle, which is crucial for optimizing surgical outcomes in reconstructive procedures. 

The primary arterial supply to the gracilis muscle is generally derived from the medial circumflex femoral artery (MCFA), although the profunda femoris and superficial femoral arteries also contribute. This vascular supply exhibits significant variability, as shown in studies by Kappler et al. [25] and Magden et al. [5]. These studies found considerable differences in the number, size, and location of the musculocutaneous perforators. Magden et al. [5] noted that the deep femoral artery was the dominant pedicle in 87% of cases, whereas Kappler et al. [25] reported that perforators were found within a specific 6 cm^2 area but varied significantly in number and diameter. 

The variability in the vascular anatomy of the gracilis muscle has direct implications for its use in reconstructive surgery. Azizzadeh and Pettijohn [2] highlighted the effectiveness of using the gracilis-free flap for dynamic facial reanimation, noting the importance of a detailed anatomical understanding to achieve excellent outcomes in facial symmetry and function. This success is contingent on the reliable identification and preservation of the MCFA and obturator nerve. 

Hallock [26,27] demonstrated the versatility of the gracilis muscle in different reconstructive scenarios, including groin wound closure and scrotal reconstruction in Fournier gangrene. His studies emphasize the importance of the consistent location of the vascular pedicles, which allows the gracilis flap to be used effectively as both a local and a free flap. These applications benefit from the muscle's reliable vascular supply, although detailed anatomical knowledge is essential to navigate the variations and ensure successful outcomes. 

Lykoudis et al. [28] and Kappler et al. [25] provided detailed anatomical studies that further our understanding of the gracilis muscle’s vascular supply. They confirmed the presence of sizable musculocutaneous perforators in a high percentage of cases, which supports the feasibility of using the gracilis perforator flap. These studies also highlight the necessity for careful preoperative planning and intraoperative flexibility due to the unpredictable nature of the perforators. 

The synthesis of these studies indicates that while the gracilis muscle offers a reliable option for various reconstructive procedures, its success heavily depends on the surgeon’s ability to adapt to its anatomical variability. Detailed preoperative planning, including the use of imaging techniques to map the vascular anatomy, and meticulous surgical techniques are critical to minimizing complications and optimizing patient outcomes. The integration of these insights into surgical practice can enhance the reliability and effectiveness of gracilis flap procedures, ultimately improving reconstructive surgery results. 

Impact on Gracilis Flap Success 

The impact of anatomical variations in the medial circumflex artery (MCA) on the success rates of gracilis flap procedures has been the subject of extensive research, with findings that highlight both the challenges and innovations in this field. Hallock [29] offers valuable insights into the techniques, outcomes, and potential complications associated with these procedures, thereby informing current practices and future directions in reconstructive surgery. 

Hallock [29] provides a historical perspective on the evolution of the gracilis musculocutaneous flap, detailing the transition from the initially unreliable vertical skin paddle to the more successful transverse orientation based on a better understanding of perforator anatomy. This study highlights the importance of preserving deep fascial connections and utilizing a transverse orientation to ensure reliable flap viability. Hallock extends these principles to demonstrate the feasibility of transferring the superior medial thigh skin as a free perforator flap based on gracilis musculocutaneous perforators alone or in conjunction with the gracilis muscle, further illustrating the versatility and reliability of this approach for wound coverage. The study reaffirms the importance of anatomical precision and innovative surgical techniques in maximizing the success of gracilis flap procedures. 

Together, these studies illustrate the nuanced relationship between anatomical variations and the outcomes of gracilis flap procedures. They underscore the importance of meticulous surgical technique, careful anatomical dissection, and innovative approaches to address the inherent variability of the MCA and its impact on flap success. By building on these insights, surgeons can enhance the reliability and effectiveness of gracilis flaps in reconstructive surgery, ultimately improving patient outcomes and expanding the applications of this versatile tissue transfer technique. 

The relationship between anatomical variations of the medial circumflex femoral artery and the success of gracilis flap procedures is complex and multifaceted, as demonstrated by the findings from recent studies. These studies contribute significantly to our understanding of how variations in vascular anatomy can impact the outcomes of such procedures, offering insights into both the challenges and solutions encountered in reconstructive surgery. 

The study by Hasen et al. [30] provides valuable insights into the challenges of obtaining a long vascular pedicle during gracilis flap procedures and introduces an extended approach to facilitate this process. They observed that this technical modification allows for safer and more efficient dissection of a longer pedicle, which is crucial for the success of free flap procedures due to the increased ease of microvascular anastomosis. Additionally, the authors highlight the rarity of complications at the gracilis donor site, advocating for the use of closed suction drainage to minimize morbidity. This study underscores the importance of surgical technique and planning in overcoming the limitations posed by anatomical variations, thereby enhancing the overall success rates of gracilis flap procedures. 

In contrast, Eom et al. [31] focus on the upper medial thigh perforator flap, noting its potential despite challenges such as short pedicle length, small vessel diameter, and inconsistent perforator positions. Their findings suggest that with a thorough understanding of the free-style free flap approach, these challenges can be mitigated. They emphasize the importance of selecting reliable perforators and tailoring the flap to the patient's needs to ensure successful outcomes. This study illustrates how intimate knowledge of vascular anatomy and innovative surgical approaches can overcome the inherent variability of the medial circumflex femoral artery and its branches, leading to successful lower extremity reconstructions. 

Hallock [32] examines the utility of the medial circumflex femoral gracilis (MCFGRACILIS) perforator free flap, highlighting its advantages such as the preservation of muscle function, reliable skin territory, and the ability to conceal the donor site scar. Despite noting potential shortcomings like flap congestion and bulkiness, the study presents the MCFGRACILIS perforator flap as a promising option for reconstructive surgery, particularly given its consistent dominant vascular pedicle. This research adds to the body of evidence supporting the versatility of gracilis-based flaps and the critical role of the medial circumflex femoral artery in ensuring their success. 

This review incorporates insights from cadaveric, imaging, and surgical studies that collectively emphasize the impact of medial circumflex artery (MCA) variations on gracilis flap procedures. Cadaveric studies, such as those by Magden et al. [5] and Kappler et al. [25], provide foundational anatomical data on the MCA's vascular pedicles, including variability in branching patterns, perforator locations, and pedicle dimensions. These studies establish the anatomical groundwork critical for both preoperative planning and intraoperative navigation. Imaging studies, exemplified by Shibuya et al. [20], underscore the importance of advanced preoperative mapping techniques, which allow for accurate visualization of MCA perforators and facilitate precise flap planning, particularly in complex reconstructions. Surgical studies, such as those by Arnež et al. [22] and Hattori et al. [23], demonstrate practical applications of anatomical and imaging insights, showcasing innovative techniques that accommodate MCA variations to optimize flap viability and reduce complication rates. These studies collectively highlight the value of a multifaceted approach, integrating cadaveric insights, preoperative imaging, and adaptable surgical techniques, to address anatomical variability and enhance the success of gracilis flap procedures. This integrative approach emphasizes the need for ongoing research to refine surgical strategies and imaging protocols, ultimately improving patient outcomes in reconstructive surgery. 

Discussion 

The relationship between anatomical variations of the medial circumflex artery (MCA) and the success rates of gracilis flap procedures represents a significant area of investigation within reconstructive surgery. This aspect of medical research is particularly crucial because it touches directly on the outcomes and well-being of patients undergoing breast reconstruction and other forms of tissue repair or replacement. The critical review of findings from the literature underscores a complex interplay of anatomical understanding, surgical precision, and patient-specific considerations. 

The studies by Natoli and Wu [19], Shibuya et al. [20], Lasso et al. [21], and others provide a panoramic view of the challenges inherent in utilizing gracilis flaps for reconstructive purposes, with a special emphasis on breast reconstruction. Natoli and Wu’s [19] findings, which report considerable variability in the vascular pedicle of the TUG flap, serve as a crucial reminder of the unpredictability that surgeons may encounter. Their revelation of a 17% variation, including instances of split and double main pedicles, underlines the necessity for rigorous preoperative planning and intraoperative flexibility. However, while their contribution enriches our understanding of pedicle variability, the study also exposes a gap in the predictive modeling of vascular variations, which can be instrumental in preoperative assessments. 

The exploration of MCA perforators' anatomical basis by Shibuya et al. [20] further extends the dialogue on the intricacies of the gracilis flap's vascular supply. Their documentation of an average of 2.4 MCFA perforators primarily passing through the gracilis muscle illuminates the anatomical variance and reinforces the importance of meticulous dissection and perforator mapping. Nevertheless, this precision in anatomical delineation raises questions about the generalizability of these findings across diverse patient populations, particularly considering body type and ethnic anatomical differences, which are not extensively discussed. 

Lasso et al.’s [21] case-based analysis contributes a vivid narrative of the challenges that unexpected anatomical variations can present during surgery. While this singular instance provides an invaluable lesson on the need for surgical adaptability, it also signals a larger issue within the field: a deficiency in extensive, large-scale studies that could provide a broader statistical basis for understanding the frequency and types of these variations. 

Furthermore, Arnež et al. [22] deliver a focused examination of the TUG flap, delineating both its advantages and limitations. The dichotomy between the potential aesthetic benefits and the practical challenges, such as flap loss and wound dehiscence, encapsulates the balancing act that defines reconstructive surgery. While the study offers detailed insights, it also accentuates the scarcity of longitudinal studies assessing long-term outcomes and patient satisfaction post-reconstruction, particularly in relation to functional and aesthetic considerations. 

The studies collectively underscore the nuanced interplay between detailed anatomical knowledge and the art of surgical adaptation. However, they also highlight several areas requiring further exploration. Notably, there is a discernible need for a more extensive data pool regarding the anatomical variations of the MCA and its implications for gracilis flap procedures. Moreover, the literature underscores a compelling need for standardized methodologies in mapping and documenting these variations to aid preoperative planning and decision-making. 

Additionally, while these studies contribute significantly to the academic and clinical understanding of gracilis flap procedures, they reveal a crucial gap in patient-centered research. Specifically, there is a notable deficiency in studies exploring patient-reported outcomes, quality of life post-surgery, and the psychological impact of donor site morbidity. These areas are essential in ensuring that the surgical approaches not only address the physical reconstruction needs but also align with the patient's overall well-being and satisfaction. 

In conclusion, the discussion drawn from the reviewed literature emphasizes the intricate relationship between MCA variations and gracilis flap success. It highlights the importance of advancing anatomical understanding, refining surgical techniques, and fostering a patient-centered approach in reconstructive surgery. Moving forward, the field could benefit significantly from integrating comprehensive anatomical studies with patient-centered research to develop more predictive and personalized surgical strategies. Such an integrative approach could help in navigating the complexities of gracilis flap procedures, ultimately enhancing patient outcomes and satisfaction in reconstructive surgery. 

## Conclusions

This systematic review highlights the critical influence of medial circumflex artery (MCA) variations on gracilis flap procedures, emphasizing the need for detailed preoperative planning and imaging to optimize outcomes. Notably, studies indicate that variations, including split and double pedicles, were often identified through preoperative imaging techniques such as CT angiography and Doppler mapping, allowing surgeons to adjust their approach to address anatomical complexities effectively. Limitations of this review include the heterogeneity of study designs and a lack of standardized imaging protocols, which may affect the comparability of results. However, strengths lie in its comprehensive inclusion of cadaveric, imaging, and surgical studies, each contributing to a nuanced understanding of the MCA’s role in flap viability. Future research should focus on developing standardized imaging and procedural guidelines to minimize complication rates associated with MCA variability, ultimately enhancing patient-specific reconstructive outcomes. 
